# Early cholangioscopy-guided lithotripsy for clearance of biliary stones associated with narrow lower bile duct

**DOI:** 10.1055/a-2552-4629

**Published:** 2025-04-04

**Authors:** Pankaj Gupta, Vikas Singla, Pankaj Singh, Kaushal Madan, Shivam Kaalia, Muzaffer Rashid Shawl, Akash Goel, Pallavi Garg, Richa Bhargava

**Affiliations:** 1 76177 Institute of Liver and Gastrointestinal Sciences, Max Super Speciality Hospital Saket, New Delhi, India

**Keywords:** Pancreatobiliary (ERCP/PTCD), Cholangioscopy, Stones, ERC topics

## Abstract

**Background and study aims:**

Stones larger than the distal common bile duct (CBD) are difficult to remove with conventional techniques. Large papillary balloon dilatation (> 12 mm) of the biliary sphincter is an effective technique for stone removal but is associated with risk of leak in patients with narrower lower CBD. Mechanical or cholangioscopy-guided lithotripsy has been used in this situation for clearance of the bile duct. In the present study, we report outcomes of early cholangioscopy-guided lithotripsy in patients with narrow lower CBD compared with stone size.

**Patients and methods:**

The present study is a retrospective analysis of prospectively collected data from all patients with large proximal stones with a narrow lower bile duct who underwent digital cholangioscopy and electrohydraulic lithotripsy (EHL). Outcomes were proportion of patients with complete bile duct clearance after the first session of electrohydraulic lithotripsy, mean number of EHL sessions for complete clearance, and complications.

**Results:**

Eighty-one patients with mean age 54 ± 17 years underwent digital cholangioscopy and EHL. Mean stone size was 14.02 ± 3.5 mm and 71 patients (87.7%) had a stone only in the bile duct. Three (3.7%) and seven patients (8.6%) had stones also in the cystic duct and intrahepatic ducts, respectively. Balloon sphincteroplasty never exceeding distal CBD size was performed in 12 patients (14.8%). Complete stone clearance was achieved in 78 patients (96.3%) after a single session. Mean number of EHL sessions were 1.04 ± 0.19. Three patients developed mild adverse events, which were managed conservatively.

**Conclusions:**

Early upfront digital cholangioscopy with EHL has high efficacy and safety for stone clearance after a single session in patients with narrow distal CBD.

## Graphical abstract

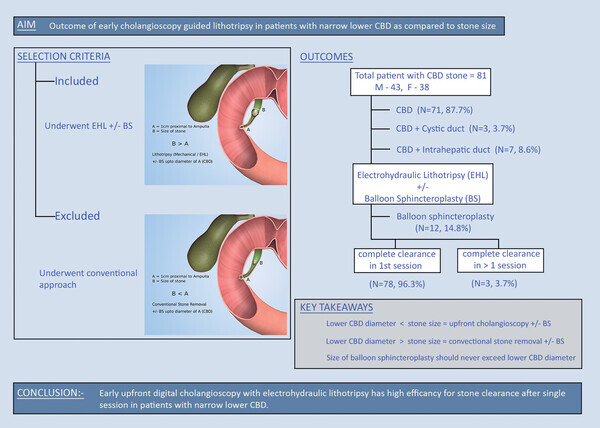

## Introduction

Gall stones affect 10% to 15% of adult population and 5% to 10% of these patients may be affected by bile duct stones 1 2 . Endoscopic retrograde cholangiopancreatography (ERCP) combined with biliary sphincterotomy followed by balloon or basket extraction has a success rate of 80% to 90% with less than 10% complications 3 . Stone size bigger than the lower common bile duct (CBD) is an important predictor of failure of ERCP and requires additional techniques 4 5 6 . Larger stones with dilated lower CBD are easier to remove than small stones with narrower lower CBD 6 . Sweeping of a stone with a balloon or basket through a narrow segment of CBD is unlikely to be successful and can lead to transmission of force in a tangential direction or impaction of the basket, respectively. Dilatation of a narrow segment of bile duct to a diameter greater than the stone or breaking the stone into pieces smaller than the lower CBD are alternate options. Endoscopic papillary large balloon dilatation is a commonly used method in patients with narrow CBD and has been advocated by European Society of Gastrointestinal Endoscopy guidelines 7 but can be associated with the complication of perforation. A large study found that balloon size larger than lower CBD to be associated with risk of perforation 8 . International guidelines suggest using a balloon that is equal to or smaller than the lower CBD 9 . The non-dilated lower segment of biliary system has two separate segments—the intraduodenal bile duct with the sphincter complex and the extraduodenal part of the bile duct 10 . Dilation of the sphincter complex and intraduodenal bile duct facilitates stone removal, but excessive stretching of the lower extraduodenal CBD may lead to leakage. Because of technical limitations, differential dilatation of the sphincter complex and extraduodenal bile duct cannot be performed, hence balloon size is limited by the size of the extraduodenal bile duct. Stones larger than the lower extraduodenal lower CBD cannot be cleared with balloon dilatation of the sphincter alone. Mechanical lithotripsy (ML) or cholangioscopy-guided lithotripsy techniques should be performed in this situation for complete clearance of the bile duct. A recent randomized controlled trial has shown higher success of cholangioscopy-guided lithotripsy over ML 11 . Evidence is emerging for high efficacy and cost effectiveness of cholangioscopy-guided lithotripsy as an early step 12 . The present study is regarding upfront usage of cholangioscopy-guided lithotripsy in patient with difficult stones which is not a standard practice at most centers. In the present study, we report outcomes of early cholangioscopy-guided lithotripsy in patients with a non-dilated extraduodenal bile duct and larger proximal stones.

## Patients and methods

The present study was a retrospective analysis of data collected prospectively at a tertiary care center between September 2021 and February 2024. Data from all patients who underwent digital cholangioscopy and lithotripsy were retrieved and analyzed. Information regarding clinical, demographic, procedure, follow-up details and complications was retrieved.

This study was approved by institute review board (BHR/RS/MSSH/DDF/SKT-2/IEC/GASTRO/24–07)

### Inclusion and exclusion criteria

All patients with a non-dilated extraduodenal bile duct with a larger stone in the upper part were included in the study. Extraduodenal CBD refers to a bile duct just before entering the duodenum. A non-dilated extraduodenal bile duct was defined as duct size smaller by 2 mm than the largest stone in the proximal bile duct. Lower CBD size was measured at 1 cm proximal to the ampullary opening. For calculation of stone size, maximum transverse diameter of the stone was measured. In case of multiple stones, the size of the largest stone was measured and considered for analysis. For measurement of CBD and stone size, fluoroscopic scale was used with scope size as reference. Pregnancy, acute cholangitis, coagulopathy, chronic liver disease, isolated cystic and intrahepatic duct stones, and age < 18 years were exclusion criteria.

### Procedure details

**Fig. 1:**
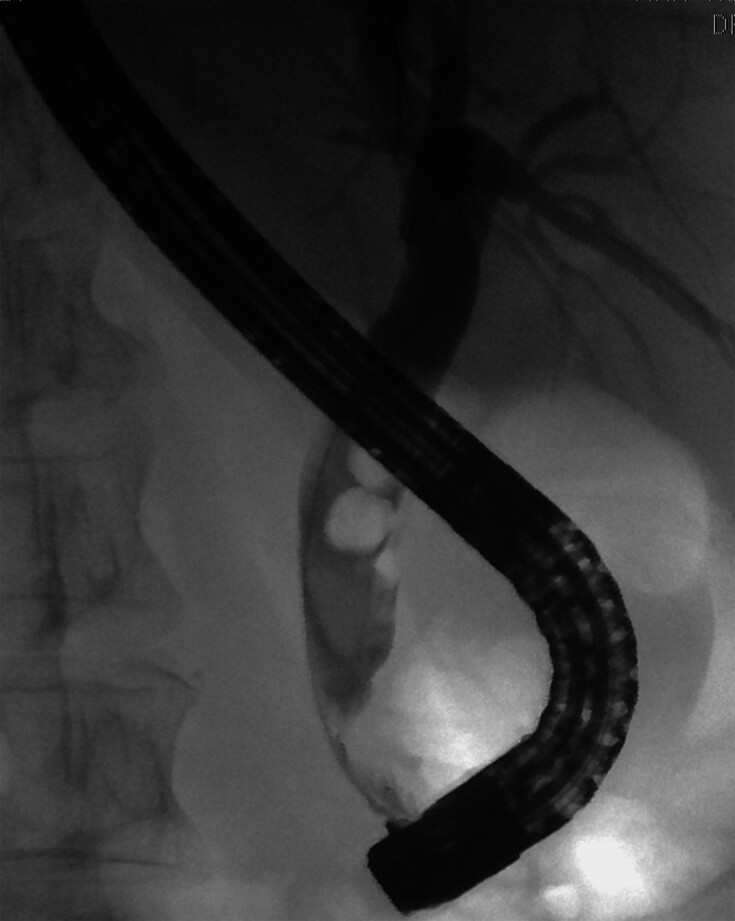
Large bile duct stones with narrow lower CBD.

**Fig. 2:**
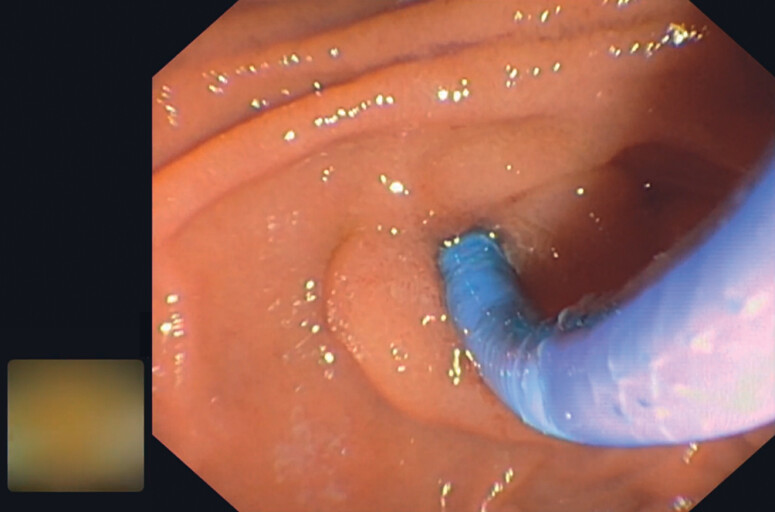
Cholangioscope insertion into bile duct.

Informed consent was obtained from all patients. All patients received a single dose of prophylactic intravenous antibiotics (third-generation cephalosporin) prior to cholangioscopy. All procedures were performed under moderate sedation with midazolam, propofol, and fentanyl in prone position. If procedure time was expected to be more than 1 hour or at anesthetist discretion, the procedure was performed under general anesthesia with endotracheal intubation. Antibiotic prophylaxis was administered in all patients at the time of the procedure. A therapeutic duodenoscope with a 4.2-mm channel (TJF Q180V, Olympus, Tokyo, Japan) was used for endoscopic retrograde cholangiopancreatography (ERCP). After CBD cannulation, a cholangiogram was obtained and the size of the lower CBD (1 cm proximal to papillary opening) and stone size were measured ( Fig. 1 ). Sphincterotomy size (Ultratome XL, Boston Scientific, Massachusetts, United States) and further balloon dilatation (Hurricane or CRE, Boston Scientific, Massachusetts, United States), was left to endoscopist discretion. If balloon dilatation was performed, balloon size never exceeded the lower extraduodenal CBD diameter, which was measured 1 cm proximal to the papillary orifice. Sphincterotomy was not extended in patients with previous sphincterotomy. Cholangioscopy was performed with the Spyglass DS system (Spyscope DS, digital controller, access and delivery catheter, Boston Scientific, Massachusetts, United States), equipped with an irrigation and aspiration system. The cholangioscope was introduced through the 4.2-mm working channel of the duodenoscope using a free-hand technique ( Fig. 2 ). In case of sharp angulation in the lower bile duct, the cholangioscope was introduced in the CBD over the wire (0.025, Visiglide, Olympus, Tokyo, Japan). We chose 0.025-inch wire because it has similar strength to a 0.035-inch wire and we did not encounter any difficulty with the 0.025-inch wire.

**Fig. 3:**
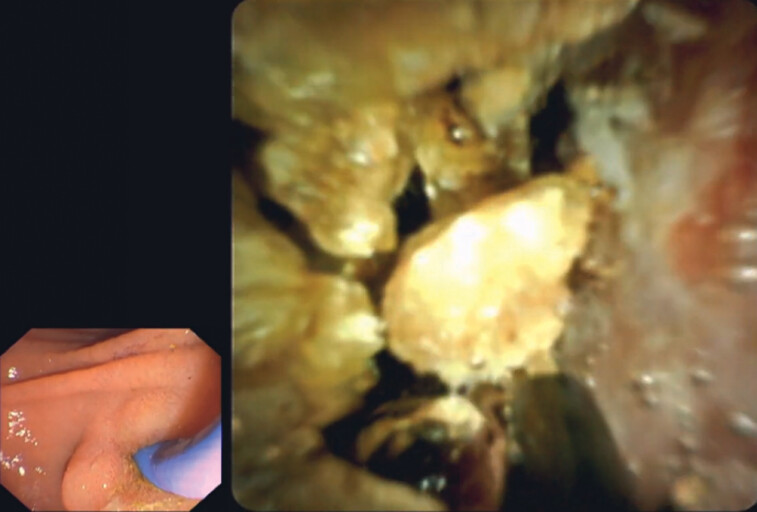
Cholangioscopy-guided electrohydraulic lithotripsy of large bile duct stones.

Electrohydraulic lithotripsy (EHL) was performed using a 1.9F bipolar electrode probe (Nortech, Elgin, Illinois, United States) with Northgate SD – 100 generator (Northgate Research Inc. Arlington Heights, Illinois, United States) ( Fig. 3 ). The energy setting was 75 volts and increased gradually to a maximum of 90 volts. Energy was applied in bursts of variable duration, at a frequency of five to six shocks per second.

Continuous irrigation was done to clear the vision field. Crushed stone fragments were removed with balloon sweep (Extractor Pro XL, Boston Scientific, Cork, Ireland), basket trawl (Dormia, Olympus, Tokyo, Japan), and if required, ML to clear floating stones, which are difficult to target with EHL. Bile duct clearance was confirmed both by cholangioscopy and cholangiogram and was followed by 7F 7-cm double pigtail stent placement in the bile duct. Stent removal was planned at 3 to 4 weeks, if the patient had improved clinically. During stent removal, a recheck cholangiogram was performed.

### Study outcomes

Outcomes of the study were the proportion of patients with complete biliary duct clearance after the first session of EHL and mean number of EHL sessions required for complete duct clearance. Complete duct clearance was defined as absence of any stone in the biliary duct and biliary radicles as confirmed with contrast injection and cholangioscopy. Occurrence of cholangitis, acute pancreatitis or bile duct leak was recorded according to the American Society for Gastrointestinal Endoscopy lexicon 13 . All continuous variables were expressed as means and standard deviation whereas categorical variables were expressed as frequencies. SPSS software version 19.0 was used for analysis.

## Results

Eighty-one patients (43 males, 38 females, mean age 54 ± 17 years) underwent digital cholangioscopy and EHL. Demographic details, baseline data for patients, and stone characteristic are shown in Table 1 and Table 2 . Fifty-seven patients (70.3%) were referred from other centers and 24 patients (30.7%) underwent primary ERCP and stone removal at our center. Mean stone size was 14.02 ± 3.5 mm, mean lower CBD diameter was 11.21, and mean lower CBD diameter and stone size ratio was 0.79. Seventy-one patients (87.7 %) had a stone only in the bile duct. Three patients (3.7%) and seven patients (8.6 %) had additional stones in the cystic duct and intrahepatic ducts, respectively. Balloon sphincteroplasty was performed in 12 patients (14.8%). Mean procedure duration was 47.5 ± 16.01 minutes.

**Table:** **Table 1** Baseline characteristics and demographic and laboratory parameters (n = 81).

Age (years), mean (SD)	54.62 ± 17.2
Male, n (%)	43 (53.1%)
Female, n (%)	38 (46.9%)
Total leukocyte count (n/mm3), mean (SD)	8.90 ± 3.97
Total bilirubin (mg/dl), mean (SD)	2.08 ± 2.73
Serum alkaline phosphate (IU/L), mean (SD)	211.38 ± 208.03
Post cholecystectomy	23 (28.4%)
Primary ERCP	24 (29.6%)
ERCP, endoscopic retrograde cholangiopancreatography; SD, standard deviation.	

**Table:** **Table 2** Procedure details and outcomes (n = 81).

Stone number	Single	35 (43.2%)
	Multiple	46 (56.8%)
Stone size	> 20 mm	3 (3.7%)
	> 15 mm	32 (39.5%)
	< 15 mm	46(56.8%)
	Mean	14.02 ± 3.5
Stone location, number (%)	Stones only in bile duct	71 (87.5%)
	Combined bile duct with cystic duct	3 (3.7%)
	Combined bile duct stones and intrahepatic stones	7 (8.6%)
Lower CBD size, number (%)	< 10 mm	69 (85.1%)
	> 10 mm	12 (14.8%)
Mean lower CBD size		11.21
Ratio of mean CBD size/stone size		0.79
Balloon dilatation		12 (14.8%)
Complete stone clearance, Number (%)	Single session	78 (96.3%)
	Second session	3 (3.7%)
Number of EHL sessions for complete clearance (mean ± SD)		1.04 ±.90
Procedure time, minutes (mean ± SD)		47.51 ± 16.03
Adverse events, number (%)		3 (3.7%)
CBD, common bile duct; EHL, electrohydraulic lithotripsy; SD, standard deviation.		

Cholangioscopic visualization of stones was successful in all the patients. Complete stone clearance was achieved in 78 patients (96.3%) after a single session. Additional ML was required in 10 patients (12.3%) for CBD clearance due to presence of floating stones, which were difficult to target with EHL. Three patients required one more additional session after 2 weeks, two of whom had a stone in both the bile duct and intrahepatic ducts and one of whom had combined bile duct and cystic duct calculi. In all three patients, complete duct clearance was achieved after the second session. The mean number of EHL sessions required for complete clearance of duct was 1.04 ± 0.19. Post procedure, two patients developed mild acute pancreatitis and one patient developed cholangitis, which were managed conservatively. None of the patients had any serious adverse events (AEs).

## Discussion

In the present study, we found high efficacy for upfront cholangioscopy and EHL in a difficult cohort of patients with narrow lower CBD and larger stones in the proximal biliary system. Rather than absolute stone size, relative stone size as compared with the lower CBD is an important parameter for success with conventional techniques To define a stone as large, we chose a difference of 2 mm compared with the lower CBD, because this was an important predictor of failure of balloon/basket-guided removal 4 . Mean stone size in our cohort was 14 mm, suggesting a difficult cohort of patients with a large stone and narrower lower end. On cholangiography, it is difficult to define the demarcation of the intraduodenal and extraduodenal bile duct. We chose a distance of 1 cm from the papillary opening to define the demarcation, as used in a previous study 14 . Balloon dilatation and lithotripsy, either alone or in combination, are commonly used in this situation. In the present study, balloon dilatation was performed in only 14.8% of patients, and was focused on dilating only the biliary sphincter. Rather than using a large balloon (≥ 12 mm) for dilatation, balloon size was guided by and never exceeded the lower CBD dimension, obviating risk of leakage. Because dilatation of the lower CBD was not performed, lithotripsy was essentially performed to clear CBD during the first session. Various techniques for lithotripsy, such as extracorporeal shock wave lithotripsy, ML, cholangioscopy-guided lithotripsy with EHL or laser, have been used previously. Although cholangioscopy-guided lithotripsy has shown to have better efficacy than ML 11 15 , patients usually undergo multiple ERCP sessions before proceeding to cholangioscopy, which increases cost, patient inconvenience, and procedure-related AEs 14 . A recent study reported a 27% reduction in number of procedures and 11% reduction in total cost of ERCPs by using cholangioscopy at an earlier stage 16 . Based on the recent literature, we performed early cholangioscopy-guided lithotripsy over ML as the first modality in our patient cohort. Benefits of cholangioscopy are direct visualization ensuring complete lithotripsy, greater efficacy for impacted stones, and confirmation of complete clearance by direct examination. We could achieve a clearance rate of 96.3% in single session, which is higher than reported with ML. Two studies previously have compared cholangioscopy-guided lithotripsy and ML. Buxbaum et al compared ML and cholangioscopy-guided lithotripsy for stones larger than 1 cm in size and found a higher success rate for cholangioscopy (93% vs 67%). Lower CBD size was not taken into consideration in the study 11 . Angsuwatcharakon et al compared mechanical and cholangioscopy-guided lithotripsy after failed balloon dilatation and found a higher success rate for cholangioscopy-guided lithotripsy (100 vs 63%); 15 of 16 patients in the cholangioscopy arm underwent large balloon dilatation. One patient had a tapering CBD and balloon dilatation was avoided 15 . Arya et al published a series of 111 patients with difficult bile duct stones using EHL and achieved a clearance rate of 90% after a single session 17 , which is comparable to results from our study. Despite selecting a difficult cohort of patients with large stones and narrow CBDs and limited usage of a large balloon, we could achieve a high success rate for CBD clearance. This can be attributed to two factors. First, participation of two operators for performing cholangioscopy and lithotripsy may improve scope maneuverability and stone targeting. Second, procedures requiring prolonged time were performed under general anesthesia with endotracheal intubation (n = 12, 14.81%).

Only three patients (3.7%) required one more session of ERCP, all of whom had an additional stone in either the cystic or intrahepatic duct. The reason for requirement of an additional session was prolongation of the procedure during the first session.

Complication rates after cholangioscopy have been reported to be higher as compared with conventional ERCP 18 . Kamiyama et al analyzed data from 44 patients who underwent EHL and reported an AE rate of 14% 19 . Arya et al, in their study, reported a post EHL complication rate of 18% 17 . In our study, the AE rate was 3.7%. Two patients developed post ERCP pancreatitis and one patient developed cholangitis, both of whom were managed conservatively. Antibiotic prophylaxis was used in all patients, and plastic stenting was placed in all patients, which may have been responsible for the lower complication rate in our study.

The strength of this study is selection of patients with difficult CBD stones with a narrow lower end, for which other modalities may not have achieved complete clearance in a significant proportion of patients or may have been associated with complications. The study also has certain limitations. Because of its retrospective design, there is the inherent limitation of missing important information. Another limitation is absence of a control arm, hence comparative analysis with alternate modality could not be performed. Lack of recurrence rate analysis is another limitation.

## Conclusions

To conclude, this study has shown high single-session success with upfront cholangioscopy for achieving complete clearance of the biliary system in patients with biliary stones larger than the lower CBD, with a low complication rate. We have proposed an algorithm for safe and effective removal of CBD stones based on our study and existing literature.
